# Efficient engagement: opportunities for mobile methadone maintenance in geographically underserved areas

**DOI:** 10.1007/s10729-026-09773-7

**Published:** 2026-06-17

**Authors:** Anthony Bonifonte, Erin Garcia, Sean Soth, Hang Ngo

**Affiliations:** 1 Data Analytics Department, Denison University, 100 West College St, Granville, OH 43023 USA; 2 Department of Industrial and Systems Engineering, Auburn University, Auburn, AL USA; 3 Evergreen Treatment Services, Seattle, WA USA; 4 Data Analytics Department (student), Denison University, Granville, OH USA

**Keywords:** Methadone maintenance treatment, Opioid use disorder, Mobile health, Access to care, Integer programming, Vehicle routing

## Abstract

In 2021 the Drug Enforcement Agency changed regulations to reduce barriers in the registration and operation of mobile methadone clinics for treating opioid use disorder in rural and underserved areas. How these routes might best be operated and how many clients might be served by mobile units are open questions. This work identifies candidate dispensing locations from mobile units in areas that do not have geographic access to a fixed clinic. Optimal routes for mobile methadone are identified by solving a mixed integer program that maximizes the number of clients without access to care that can be served by a mobile route. Ohio is used as a case study state to explore the efficiency of routes leaving from each methadone clinic in the state. The approach is generalized nationwide, and multi-vehicle routes are analyzed. Between 48 and 68 clients can be served by a single vehicle in a day (mean of 60) under default parameter assumptions across Ohio. Similarly, nationwide routes serve a mean of 47.5 clients per day. The second and third vehicle routes from a clinic provide access to an additional 95% and 93% of the clients of single vehicle routes, respectively. The number of servable clients on any route greatly differs based on geography. This work demonstrates the potential value of mobile methadone maintenance in reaching populations without geographic access to a fixed clinic. Additional policy and funding changes are needed to incentivize the operation of mobile methadone units for serving rural and unmet demand.

## Highlights

Mathematical models can help plan mobile methadone routes to serve new clients.

Nationwide, mobile routes can serve 47 new clients on average.

Number of clients per day is more dependent on service time than setup time.

Optimal routes primarily stop only once on the boundary of current coverage areas.

Mobile routes are not financially sustainable under current funding models.

## Introduction

Opioid misuse and dependence is a nationwide public health crisis. In 2019, opioid overdoses resulted in 49,860 deaths in the United States [1] and an estimated 10.1 million people aged 12 years or older misused prescription opioids [2]. The total estimated economic burden of the use of opioids for non-prescribed purposes was $78.5 billion in 2013 [3].

Among the many treatment protocols available to those with opioid use disorder (OUD) is medication for opioid use disorder (MOUD). The Federal Drug Administration (FDA) has approved only three medications under this category of treatment: buprenorphine, naltrexone, and methadone. Methadone is a synthetic opioid that blocks the euphoric effects of other opioids [4] and has been shown to reduce opioid dependence and overdoses. In most common use cases, methadone treatment has higher retention than buprenorphine [5] and is only dispensed in one-day supply at federally certified, accredited Opioid Treatment Programs (OTPs), also referred to as methadone clinics. Individuals accessing methadone typically remain on the treatment for months or even years [6, 7], so geographic access to these clinics is a key factor in their utilization and effectiveness.

In 2021, the Drug Enforcement Administration (DEA) changed long-standing regulations on the operation of mobile narcotic treatment programs, also known as mobile clinics, to increase access to methadone in rural areas [8]. Prior to this change, mobile clinics had to be registered as separate treatment facilities, creating expensive and onerous barriers to mobile clinic operation. The rule change eased regulations to simplify registration and operation of these mobile units out of existing fixed brick-and-mortar methadone clinics. Two of the key requirements are they must begin and end each day at the same registered home clinic, and routes must remain within the home state.

Many works have identified challenges in providing care to underserved areas, including geographic distance, limited medical facilities, and gaps in infrastructure [9]. Specific efforts have attempted to encourage greater availability of MOUD in rural areas [10, 11]. Works have highlighted the potential for mobile methadone clinics to enhance retention in care and provide access for underserved populations [12, 13]. One study in Baltimore found mobile methadone units quadrupled retention in care compared to fixed methadone clinics [14]. Other cohort studies have reported successful operations of mobile methadone units in urban areas [15–18]. More broadly, mobile health clinics have had positive community health effects [19]. The potential impact of adding new mobile routes to serve new clients identified at a zip code level in Louisiana was analyzed using simulation [20], highlighting the benefit of using modeling tools when selecting service areas.

Vehicle routing problems are commonly used in health delivery systems and are modified to account for the requirements within specific contexts. Variants have been used in blood collection and distribution systems, including fixed networks where sequencing and timing must be determined [21], network design that incorporates routing decisions [22], and selecting stops for mobile blood collection with routing of both the collection and transport vehicles [23]. Mobile health care routes have also been studied in prenatal care in rural areas in Romania [24], mobile mammography units [25], and home health care delivery [26]. Our work builds on these studies in the context of OUD treatment for those without current geographic access to care. One unique contribution of this study is incorporating both client and provider constraints in a daily care setting.

One author of this study works for a Washington-based OTP that currently operates multiple mobile medication units across the state. Based on the practical constraints, workflows, and regulatory considerations encountered in that organization’s implementation, we develop this proof-of-concept study that creates a general framework for clinics to use. We propose a mathematical model to identify optimal routes for mobile clinics operating out of fixed methadone clinics. This work begins with a case study of Ohio, explores sensitivity analyses, then generalizes nationwide in the United States. We demonstrate the large potential gains from operating mobile clinics and examine characteristics related to their operation. To our knowledge, this is the first study to examine the problem of identifying locations to stop mobile methadone clinics and constructing routes using optimization models. Our model is intentionally designed so that any OTP can adapt it by using locally available data such as population densities, parking feasibility, road access, and anticipated daily patient volume.

In Sect. 2 we identify data sources for parameterizing our models, including geographic, methadone clinic, and pharmacy data. In Sect. 3 we describe our model for identifying optimal stops and routes for mobile clinics. In Sect. 4 we present main results and sensitivity analyses for key model parameters. In Sect. 5 we discuss the implications of this study on operation of mobile routes. Finally, in Sect. 6 we provide limitations and suggestions for future work.

## Data collection

All data, code, and supplementary results are available at https://github.com/bonifontea/Mobile-Methadone.

### Data sources

As of the time of submission for this manuscript, data collection from many of the sources below had not yet resumed post-Covid. Because our work explores the relationship between populations, drug use, and availability of methadone maintenance treatment, it is important that all data reflect the same point in time. We therefore chose to utilize the most recent pre-2020 data from all sources.

#### Methadone clinics and utilization

We collect existing outpatient methadone clinic locations and state level methadone usage from the National Survey of Substance Abuse Treatment Services (N-SSATS) 2019 directory administered by the Substance Abuse & Mental Health Services Administration [27]. Nationwide, 401,684 methadone clients are served across 1,424 clinics (an average of 282 clients per clinic). Connecticut, Delaware, Rhode Island, and Washington DC have no unmet demand (based on criteria described below) and therefore are not eligible for mobile routes since routes are restricted to a single state. Additionally, ten clinics in Arizona and Massachusetts cannot feasibly serve any unmet demand with a mobile clinic. Removing these clinics leaves 1,350 candidate clinics to use as origins of new mobile routes. Each clinic is classified as metropolitan, micropolitan, small town, or rural, based on the census-tract level primary Rural-Urban Commuting Area (RUCA) Codes [28].

In this work we assume clients are able to travel up to *r* minutes to reach a stopped mobile methadone clinic. Beyond just capability and willingness to travel, this maximum travel time accounts for additional restrictions clients may face in attending a mobile clinic, such as time-of-day limitations. Therefore larger values of *r* represent fewer restrictions on clients, and smaller values of *r* represent more restrictions (such as difficulty taking time off work when the clinic is available, or lack of public transportation). The Department of Health and Human Services State Standards for Access to Care in Medicaid Managed Care publishes guidelines for acceptable limits of travel distance to care [29]. The guidelines vary state to state and are subdivided by primary care vs. specialist care, with significantly shorter allowable drive distances to primary than specialist care. To reflect patient need to travel daily to receive care, we treat MOUD as primary care. In the base case we let *r* = 25 min consistent with these guidelines for primary care; we also explore *r* = 10 and *r* = 5 min to assess the sensitivity of the routes to changes in this parameter.

#### Pharmacies

We collect locations of pharmacies from RxOpen.org, a tool developed by Healthcare Ready, which lists all prescription pharmacies and dialysis centers within the United States [30]. There are 2,150 pharmacies within Ohio and 61,332 nationwide.

#### Census tracts and populations

We gather census tract centroids from the US Census Bureau (2012) and population counts from the 2019 American Community Survey 1-Year Estimates [31]. Each census tract has between 1,200 and 8,000 people, with a nominal size of 4,000 people. Ohio has 2,940 census tracts, with 83,616 nationwide. Census tract centroids are defined as the mean center of population within each tract based on the 2010 census.

#### Road maps and drive distances

We collect road maps from the OSMnx package in Python [32]. This package queries datasets maintained by OpenStreetMap, an international not-for-profit organization providing freeware maps of the world [33]. The Nominatim tool within OpenStreetMap Addresses geocodes addresses to longitude and latitude. The NetworkX package in Python [34] calculates road travel times between census tract centroids and pharmacies, methadone clinics and pharmacies, and each pair of pharmacies. This package treats road maps as graphs, where clinics and pharmacies are nodes and roads are edges. It calculates drive distances and corresponding drive times by solving the shortest path problem between each location pair using Dijkstra’s algorithm [35].

#### Operational parameters

The default parameter values are based off operational considerations, which are influenced by DEA regulations. For example, regulations around medication storage necessitate certain procedures which require setup time at each stop. We set default parameters of *h* = 7 working hours, *r* = 25 min allowable drive time, *t* = 60 min setup time, and *s* = 5 min service time. One author on this study, a director at an OTP, has confirmed these align with their current operations.

### Estimating unmet demand

A fundamental challenge in planning new mobile routes for methadone maintenance treatment is identifying potential locations that provide access to those seeking treatment services. In areas with no history of methadone clinics, it is difficult to estimate how many individuals would utilize the service. Our prior work addresses this challenge and provides a methodology to predict the number of individuals that would initiate methadone treatment if geographic access restrictions were removed [36]. In summary, we build a nationwide simple regression model predicting state-level methadone utilization based on survey-driven estimates of heroin usage. The state-level estimate of unmet methadone demand is then distributed to census tracts without geographic access to a fixed methadone clinic proportional to heroin usage. When implementing a new mobile route in practice, specific outreach initiatives could refine these estimates.

### Pharmacy clustering to identify candidate stops

The pharmacy identification and clustering process is completed individually within each state. We describe the steps in the context of the state of Ohio. As mentioned in Sect. 2.2, we identify 2,150 pharmacies in Ohio as possible stopping locations for our mobile methadone clinics. A subset of these pharmacies is selected as the set of usable stops for the mobile route instead of the full set for multiple reasons. First, it is unreasonable to allow a mobile clinic to stop at several closely adjacent locations. Given that the primary purpose of the mobile clinics is to serve unmet demand in predominantly rural communities, traveling a small distance away is counterproductive, as many clients would share access to both locations. Second, pharmacies are acting only as a proxy for areas with a reasonable infrastructure and density of residents (i.e. not in the middle of farmland), and therefore selecting many stops in a small area is unnecessary. Finally, maintaining the full list of pharmacies renders the model computationally infeasible. As we are using a mixed integer programming model, the run time is highly sensitive to the number of integer variables. Attempting to solve the routing problem with all 2,150 pharmacies in the state as potential stops creates 2,150^2^ ~ 4.6 million binary variables as defined in the model below, and a single instance of the problem is not solvable within 8 hours run time. Using the clustering algorithm described in the following paragraph reduces the number of pharmacies to 201. With only 201^2^ ~ 40,000 binary variables, every model instance can be solved to optimality in under 1 s.

**Fig. 1:**
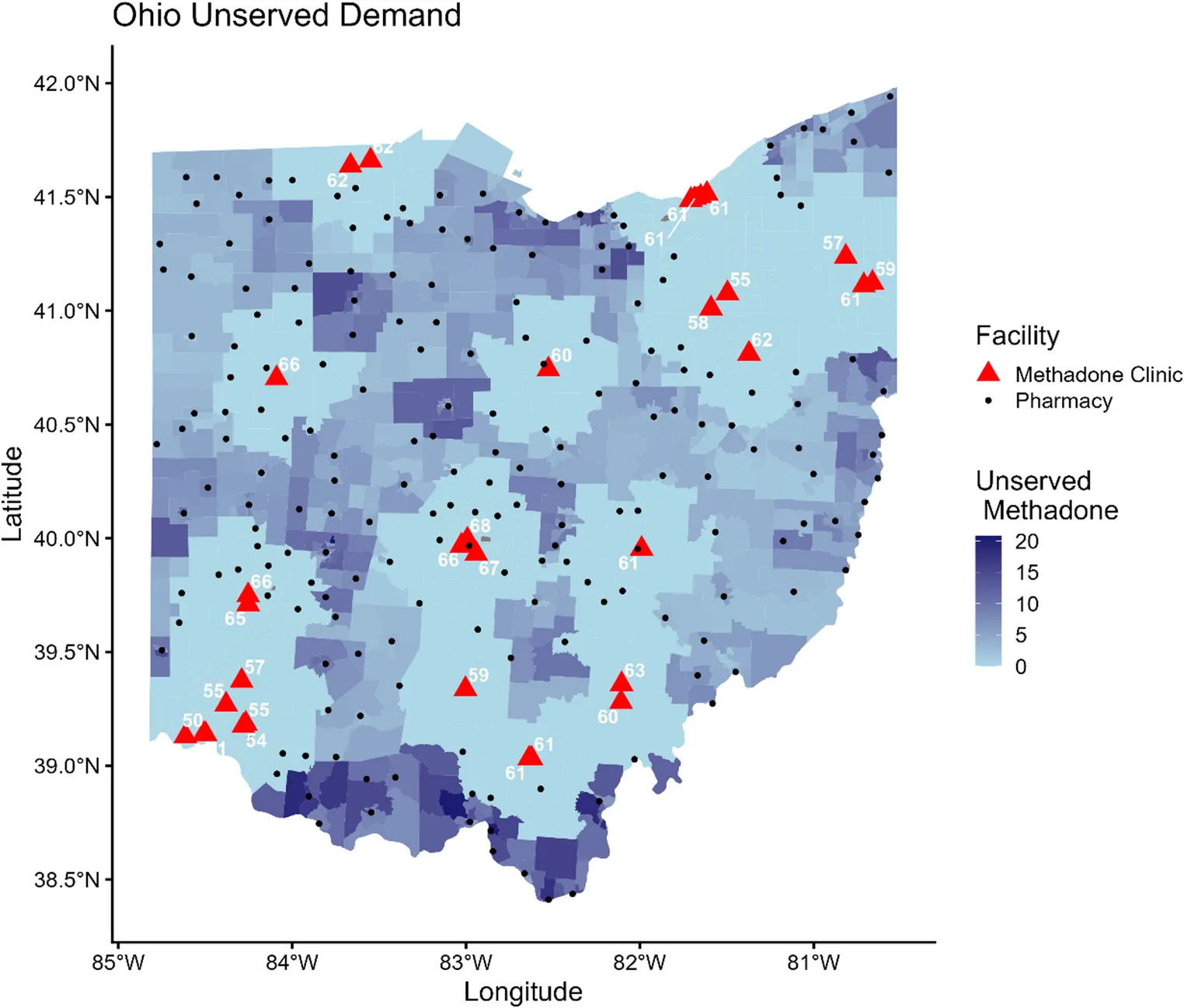
Ohio pharmacies, existing methadone clinics, and unmet methadone demand. Red triangles are existing methadone clinics, white numbering near each clinic triangle is the amount of demand served by the optimal route originating from that clinic under default parameter values, and black dots are potential pharmacy stops

To reduce the set of pharmacies representing candidate stops, we use a leader cluster algorithm to create clusters of closely located pharmacies [37]. The leader cluster algorithm requires the approximate radius of a cluster as a tuning parameter, which we take to be 8.05 km (5 miles). The resulting clusters are made up of a central location and all pharmacies within an 8.05 km radius of this center. The algorithm creates 312 pharmacy clusters in Ohio, and we take the pharmacy which is closest to the central point to be a candidate stopping location for the mobile route. We allow all demand within *r* minutes of any pharmacy in the cluster to be served by the central pharmacy to ensure this clustering does not unnecessarily restrict access. Next, we remove pharmacy clusters that do not have any unmet demand within *r* minutes; these are primarily located in urban areas. Finally, we remove pharmacy clusters which are strictly dominated, in the sense that all servable demand has shorter travel time to a different pharmacy cluster. This results in 201 pharmacy clusters, each represented by one central pharmacy, spread across the state. These pharmacies, existing methadone clinics, and the unmet demand at a census tract level are shown in Fig. 1.

## Optimization model of mobile clinic stops and routes

In this section we describe a Mixed Integer Linear Program (MILP) that solves for optimal routes for mobile methadone clinics to serve as many clients as possible. Table 1 provides the notation and variables used in this model. The optimization model is given by the following:

**Table 1:** Notation and decision variables in this study

**Set Notation**	
*C*	Set of census tracts with currently unmet demand
*M*	Set of existing methadone clinics
*P*	Set of pharmacies
*L* = *M* U *P*	Set of all locations
*V*	Set of vehicles operating from clinic
**Data**	
*m*_*i*_	Methadone demand in census tract *i; i* ϵ *C*
*d*_*i,j*_	Drive time from location *i* to location *j; i*,*j* ϵ *L*
*g*_*i,j*_	Drive time from census tract *i* to pharmacy *j*; *i* ϵ *C*, *j* ϵ *P*
**Parameters**	
*r*	Allowable time a client can travel to reach a mobile clinic
*s*	Fixed setup time at for each pharmacy stop
*t*	Service time per customer
*h*	Available hours of operation in day
*f*	Fixed clinic the route is based out of
*a*	Arbitrary large number (for modeling purposes)
**Decision Variables**	
*x*_*i,j,v*_	1 if vehicle *v* travels from location *i* to location *j*, 0 if not; *i*,* j* ϵ *L*, *v* ϵ *V*
*y*_*j,v*_	1 if vehicle *v* stops at pharmacy *j*, 0 if not; *j* ϵ *P*,* v* ϵ *V*
*z*_*i,j,v*_	How much demand from tract *i* is served at stop *j* by vehicle *v; i* ϵ *C*, *j* ϵ *P*,* v* ϵ *V*

Decision Variables:

- *x*_*i,j,v*_ = {1 if vehicle *v* travels from location *i* to location *j*, 0 if not}; *i*,* j* ϵ *L*,* v* ϵ *V*.
- *y*_*j,v*_ = {1 if vehicle *v* stops at pharmacy *j*, 0 if not}; *j* ϵ *P*,* v* ϵ *V*.
- *z*_*i,j,v*_ = How much demand from tract *i* is served at stop *j* by vehicle *v*; *i* ϵ *C*, *j* ϵ *P*,* v* ϵ *V*.

Objective:

$$\:max{\sum\:}_{i\in\:C}^{}{\sum\:}_{j\in\:P}^{}{\sum\:}_{v\in\:V}^{}{z}_{i,j,v}\:\:\:$$

Subject to:

1 $$\:{\sum\:}_{j\in\:P}^{}x_{f,j,v}=1\:\forall\:v\in\:V$$

2 $$\:{\sum\:}_{i\in\:P}^{}x_{i,f,v}=1\:\forall\:v\in\:V$$

3 $$\:{\sum\:}_{i\in\:L}^{}x_{i,k,v}=\:{\sum\:}_{j\in\:L}^{}x_{k,j,v}\:\forall\:k\in\:L;\:\forall\:v\in\:V$$

4 $$\:y_{k,v}={\sum\:}_{i\in\:L}^{}x_{i,k,v}\:\forall\:k\in\:P;\:\forall\:v\in\:V$$

5 $$\:z_{i,j,v}\leq\:ay_{j,v}\:\:\forall\:i\in\:C\:;\:\forall\:j\in\:P;\:\forall\:v\in\:V$$

6 $$\:{\sum\:}_{v\in\:V}^{}{\sum\:}_{j\in\:P}^{}z_{i,j,v}\leq\:m_i\:\:\:\forall\:i\in\:C$$

7 $$\:z_{i,j,v}=0\:\:\:\forall\:i,j\in\:L:\:g_{i,j}>r;\:\forall\:v\in\:V$$

8 $$\begin{array}{c}\:{\sum\:}_{i\in\:L\:}^{}{\sum\:}_{j\in\:L}^{}d_{i,j}x_{i,j,v}+s{\sum\:}_{j\in\:P}^{}y_{j,v}+\\t{\sum\:}_{i\in\:C}^{}{\sum\:}_{j\in\:P}^{}z_{i,j,v}\leq\:h\:\:\:\forall\:v\in\:V\:\end{array}$$

9 $$\:{\sum\:}_{i\in\:S}^{}{\sum\:}_{j\in\:S}^{}x_{i,j,v}\leq\:\left|S\right|-1\:\:\forall\:S\subset\:L;\forall\:v\in\:V$$

10 $$\:z_{i,j,v}\geq\:0,\:{\:\:\:\:y}_{i,v},x_{i,j,v}\in\:\left\{\mathrm{0,1}\right\}\:\forall\:i,j\in\:L;\:\forall\:v\in\:V$$

The notation *C* denotes the set of census tracts with currently unmet demand, *M* denotes the set of fixed methadone clinics, *P* denotes the set of pharmacies, and *V* the set of vehicles (routes). The set of all locations including both clinics and pharmacies is denoted *L*. The objective function maximizes the amount of unmet demand met by the stops along the mobile route. Constraints (1) and (2) require that each mobile route must leave and enter the designated fixed clinic *f*, ensuring that the routes begin and end their day at that clinic. (3) ensures if the mobile clinic enters a location it must also leave, ensuring continuity of the route. (4) says a stop can only be made at a pharmacy if the route visits that pharmacy. (5) ensures demand can only be met at a pharmacy if the mobile route stops there. Parameter *a* is an arbitrarily large number which forces demand served at a pharmacy to be 0 if no stop is made (*y*_*j,v*_ = 0). (6) says the combined demand from a census tract served by all pharmacies and vehicles cannot be greater than the demand in that census tract *m*_*i*_. (7) restricts client travel time *g*_*ij*_ to a mobile unit to a maximum of *r*. (8) limits the hours of operation to the available time *h* in the day, considering drive time *d*_*ij*_, setup time *s*, and service time *t*. (9) are the subtour elimination constraints, ensuring one continuous route is created and not multiple smaller routes. These constraints are standard for vehicle routing problems and are added dynamically as the MILP solves. Finally, (10) ensures nonnegativity of demand served and defines the route and stop variables as binary.

## Results

### Single vehicle routes

We first consider the specific case with only one vehicle that traverses the same route every day. Owing to large start-up costs, many clinics only operate a single mobile unit (personal communication), and clinics looking to initiate a mobile program would likely begin with a single unit.

#### Ohio results

**Fig. 2:**
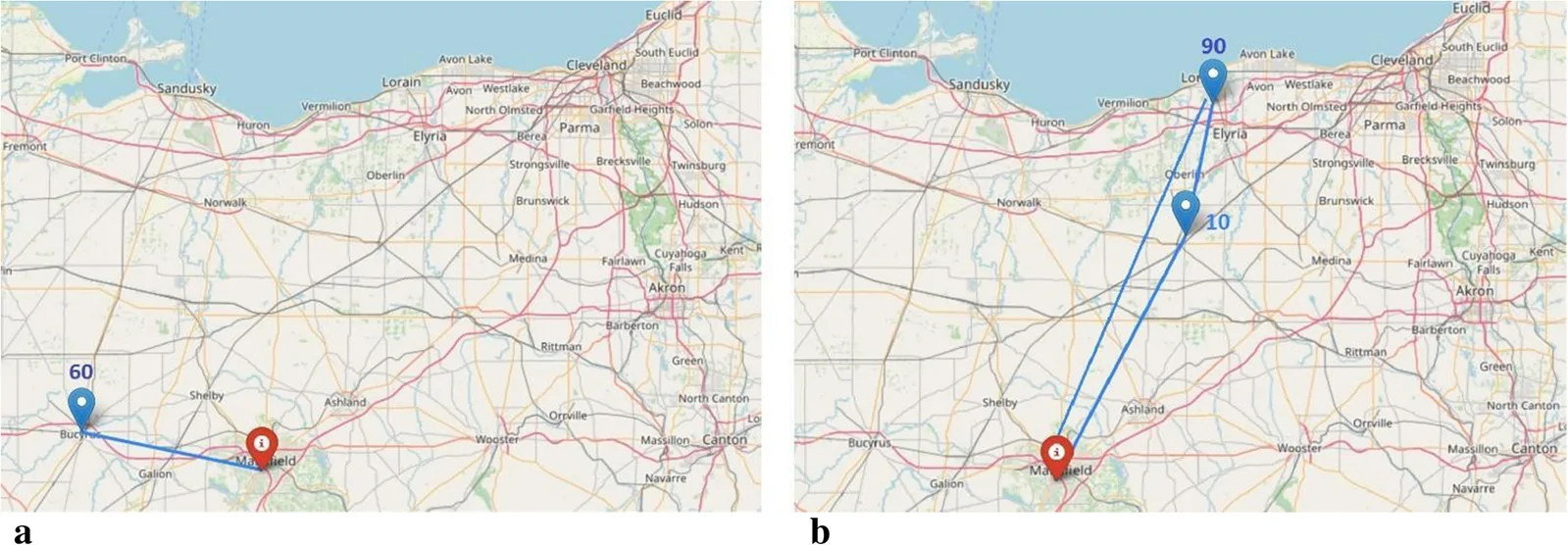
Mobile clinic route based out of Mansfield Ohio. The red markers indicate the fixed methadone clinic (the starting and stopping location of the mobile route). The blue markers are stops along the route, and the text next to each stop is the number of clients served at that stop. **2a**: service time 5 min, setup time 60 min, max travel 25 min. **2b**: service time 2.5 min, setup time 15 min, max travel 5 min

We explore 32 routes originating from each fixed clinic across Ohio under the default parameter settings described in Sect. 2.1.5. Routes across the state are able to serve a moderate number of clients in one day, ranging from 48 to 67 per route, with a mean of 59.7 (sd = 4.8). Every mobile route makes only a single stop and has an average round trip drive time of 1.02 hours (sd = 0.40). This suggests that, in general, mobile routes will likely travel a short distance from their origin clinic and make relatively few stops, and that the demand is sufficient to occupy more than a full day’s service at these stops 02 (Fig. 2).

Based on the RUCA Code classification, 27 of Ohio’s 32 clinics are in metropolitan census tracts, and the remaining 5 clinics are located in micropolitan ones (none of the existing clinics are located in small town or rural tracts). Routes originating from clinics in micropolitan tracts are able to serve marginally more demand than ones based out of metropolitan clinics (60.9 vs. 59.5) with very similar total drive times (0.93 vs. 1.03 hours), although neither difference was statistically significant.

#### Ohio sensitivity analyses

**Fig. 3:**
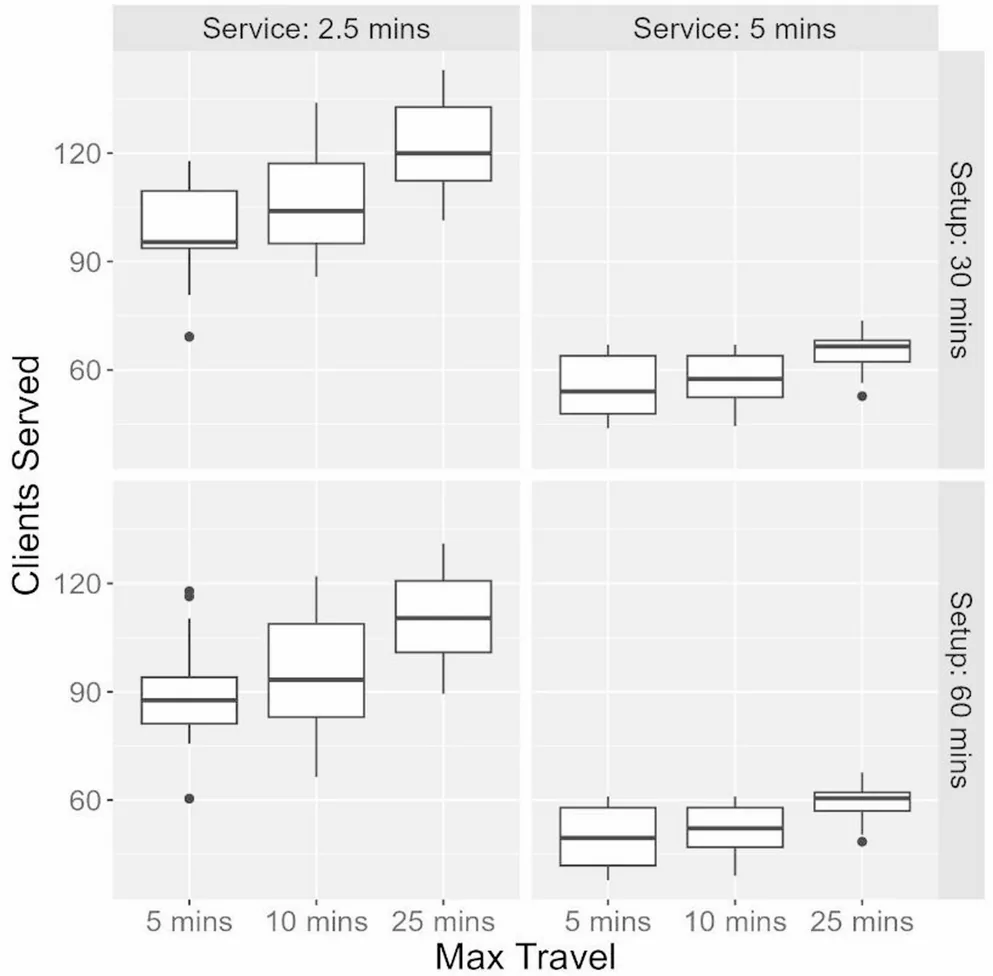
Sensitivity analysis of customers served as a function of key model parameters

Figure 3 shows the number of clients served as a function of several key model parameters. A change in a single model parameter can occur through several different operational modifications. For example, a decrease in the setup time model parameter can be the result of an actual reduction in the time that it takes to complete the setup task. Or, a change in the process could allow some of the setup tasks to occur while the vehicle is in transit, so the time required after a vehicle arrives at the service location decreases. Similarly, halving the service time parameter can be the result of either decreased service time per individual customer or doubling the number of available dosing stations.

As expected, the number of servable clients increases as each of the operation times decreases, although not by similar proportions. For example, compared to the base case when the service time *t* equals 5 minutes and the setup time *s* equals 60 minutes (mean 59.7 clients served), halving the service time to 2.5 minutes results in an average of 109.9 clients served per route, an 84% increase. Setup times, however, have a relatively small effect on the number of clients served. For example, fixing the service time to 5 minutes and halving the setup time from 60 minutes to 30 minutes only results in an average of 5 additional clients being served each day, despite every route continuing to have only 1 stop. Finally, assuming clients cannot travel as far to reach the mobile clinic decreases the number of servable clients in a day, as would be expected. Assuming clients can travel a maximum of 10 minutes to reach the mobile clinic while keeping the other parameters at baseline values results in an average of 51.8 clients served, a reduction of 7.9 clients per route from the base case. Under most parameter values the number of stops the mobile clinic makes is only 1. In the most extreme case when the service time *t* equals 2.5 minutes, the setup time *s* equals 30 minutes, and client maximum travel is 5 minutes, routes still only make 1.38 stops on average.

To illustrate features of the optimal solutions, consider Fig. 2a, which displays the planned route based out of Mansfield, a rural area in north-central Ohio, under the base case parameters. This route drives 29 min west to the town of Bucyrus, serves 60 of the possible 72 clients at that location in the time available, and then returns to the clinic. Small decreases in the service and setup time parameters allow more clients in Bucyrus to be served on this same route. Figure 2b shows the route originating from the same Mansfield clinic when the model parameters are significantly reduced. In this case, the service time is 2.5 min, the setup time is 15 min, and the maximum travel time a client can have is 5 min. The optimal route travels north 47 min to serve 10 clients in Wellington, then continues north an additional 23 min to serve 90 clients in Lorain before returning to the clinic. This example shows how changing the model parameters impacts not only how many clients can be served at each location, but also which candidate stops are selected at all. The differences in this example highlight the intuitive differences that result from changing the parameter values. If the setup and service times are longer, the model cannot choose to visit locations farther away from the clinic even if there is high demand because there will not be enough time to serve those customers. But when the service and setup times are smaller, it is possible for the mobile route to serve enough people at a more distant location to offset the longer drive time.

#### Nationwide results

We expand our analysis to consider 1,350 routes originating from each fixed clinic nationwide. These routes are generated using the default parameter settings of working *h* = 7 hours, allowable drive time *r* = 25 min, setup time *t* = 60 min, and service time *s* = 5 min. As in the above sections, each clinic dispatches a single vehicle that takes an identical route every day. Optimal routes serve an average demand of 47.5 (sd = 12.5), spend 1.73 hours a day driving (sd = 0.69), and make 1.13 stops in a day (sd = 0.33). No route makes more than two stops per day. Similar to Ohio results, this indicates mobile units spend a considerable amount of time driving to reach unmet demand and overwhelmingly tend to only have a single stop.

We conduct a sensitivity analysis to explore whether the optimal routes are unique or if other available routes could serve a similar number of clients. Because this problem is a mixed integer program, reduced costs are not defined as they would be for a linear program. Therefore we explore the performance of different routes by forcing individual candidate pharmacy stops to be included one at a time in separate models. For each starting clinic nationwide, a new model for each reachable pharmacy was solved individually with a constraint requiring that pharmacy to be included on the route. Across these models, we find 42.1% of clinics do not have alternate routes that can serve within 5% of the optimal demand. For the other 57.9% of clinics, the average number of near-optimal alternatives is 2.03.

**Fig. 4:**
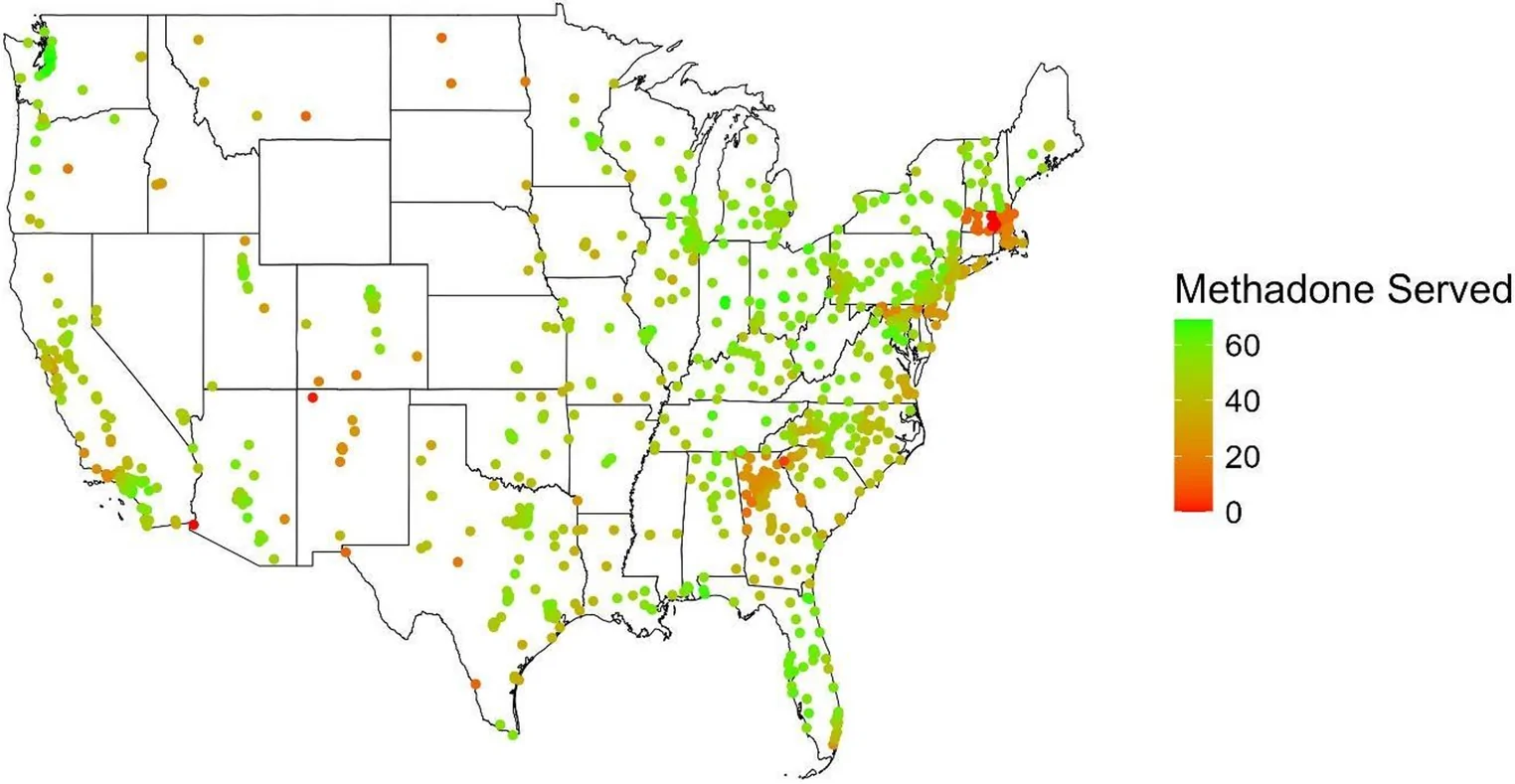
Nationwide map of unmet demand serviceable from existing clinics. Default parameter values setup = 60 min, service = 5 min, maximum travel = 25 min

Figure 4 shows a map of how much demand can be served by a mobile unit operating out of each clinic nationwide. This map demonstrates significant variation in how much demand can be served, reflecting both density of clinics and abundance of unserved demand. For example, clinics in Atlanta, Georgia can only serve between 10 and 30 clients in a day, owing to the high density of clinics and their distribution throughout the city which results in the nearest unserved demand being more than 30 miles away. The route originating at one specific clinic in the center of Atlanta must drive over 1 hour and 45 min each way to reach any unserved demand. However, clinic density does not by itself render operations ineffective. Within the city of Los Angeles, California, clinics on the south side of the city are able to serve approximately 60 clients in a day, while those on the north side of the city can serve only 30. Both Seattle, Washington and Chicago, Illinois have a high density of clinics, yet each are able to serve over 60 clients in a day. The low population density in some states such as Idaho, Montana, North Dakota, and New Mexico leave mobile routes only able to serve approximately 20 clients in a day.

Table 2 shows the nationwide results broken down by the urbanicity of origin clinic. Of the 1,350 clinics that are used as starting points for mobile routes, 1,207 (almost 90%) are located in census tracts that are designated as metropolitan. These routes can serve an average of 47.7 clients per day and have an average drive time of 1.72 hours. This is marginally more clients and less drive time than is achieved with routes from clinics in micropolitan (108 routes) and small town tracts (29 routes), which serve 46.1 and 45.2 clients and have drive times of 1.8 and 1.9 hours per day, respectively. These results differ slightly from those seen in Ohio, which saw slightly decreased route performance out of micropolitan clinics compared to metropolitan ones. The six mobile routes originating from clinics in rural areas, however, can be assigned to serve an average of 48.7 people per day and only have an average drive time of 1.6 hours. One of the six rural area routes is in such a sparsely populated area that the optimal single vehicle route is only able to serve 22.6 people with two different stops on the route and a drive time of 2.3 hours. Without this route, the difference in the reach of a single mobile route from a rural clinic compared to the other RUCA designations becomes more pronounced. The average number of clients served by the remaining rural mobile routes is 54, and the average drive time is reduced to 1.5 hours.

**Table 2:** Summarized results of baseline model. Numbers in parentheses indicate standard deviation

Clinic Type	*n*	Demand Served	Drive Time, hours
All	1,350	47.5 (12.5)	1.7 (0.7)
Metropolitan	1,207	47.7 (12.6)	1.7 (0.7)
Micropolitan	108	46.1 (10.8)	1.8 (0.7)
Small town	29	45.2 (8.9)	1.9 (0.7)
Rural	6	48.7 (7.8)	1.6 (0.6)

### Multi-vehicle routes

In this section we consider the case study of Ohio clinics operating two or three mobile routes. In addition to the default scenario of a clinic operating multiple vehicles that serve distinct customers on daily routes, this scenario also models the situation where a clinic operates a single vehicle that dispenses multi-day take-home doses. For example, a clinic that operates one vehicle on two alternating daily routes, providing same-day and one take-home methadone dose, is equivalent to a two-vehicle problem. Similarly, operating three alternating daily routes each with same-day and two take-home doses is equivalent to a three-vehicle problem. This provides greater capacity to serve unmet demand in potentially disparate geographic areas if regulatory and safety measures can be addressed.

A second vehicle operating from each clinic is able to serve between 84.9% and 100% of the demand of the optimal single-vehicle route from each clinic, with an average of 95.5%. Put another way, the average demand met with two vehicles was 116.7 across Ohio clinics, compared to 59.7 for single vehicle routes. Third vehicles were able to serve between 76.9% and 100% additional demand of the optimal single-vehicle route, with an average of 92.6%. The average demand met with three vehicles was 172.1 across all clinics. These findings highlight that there is sufficient demand near most clinics to support multiple routes.

**Fig. 5:**
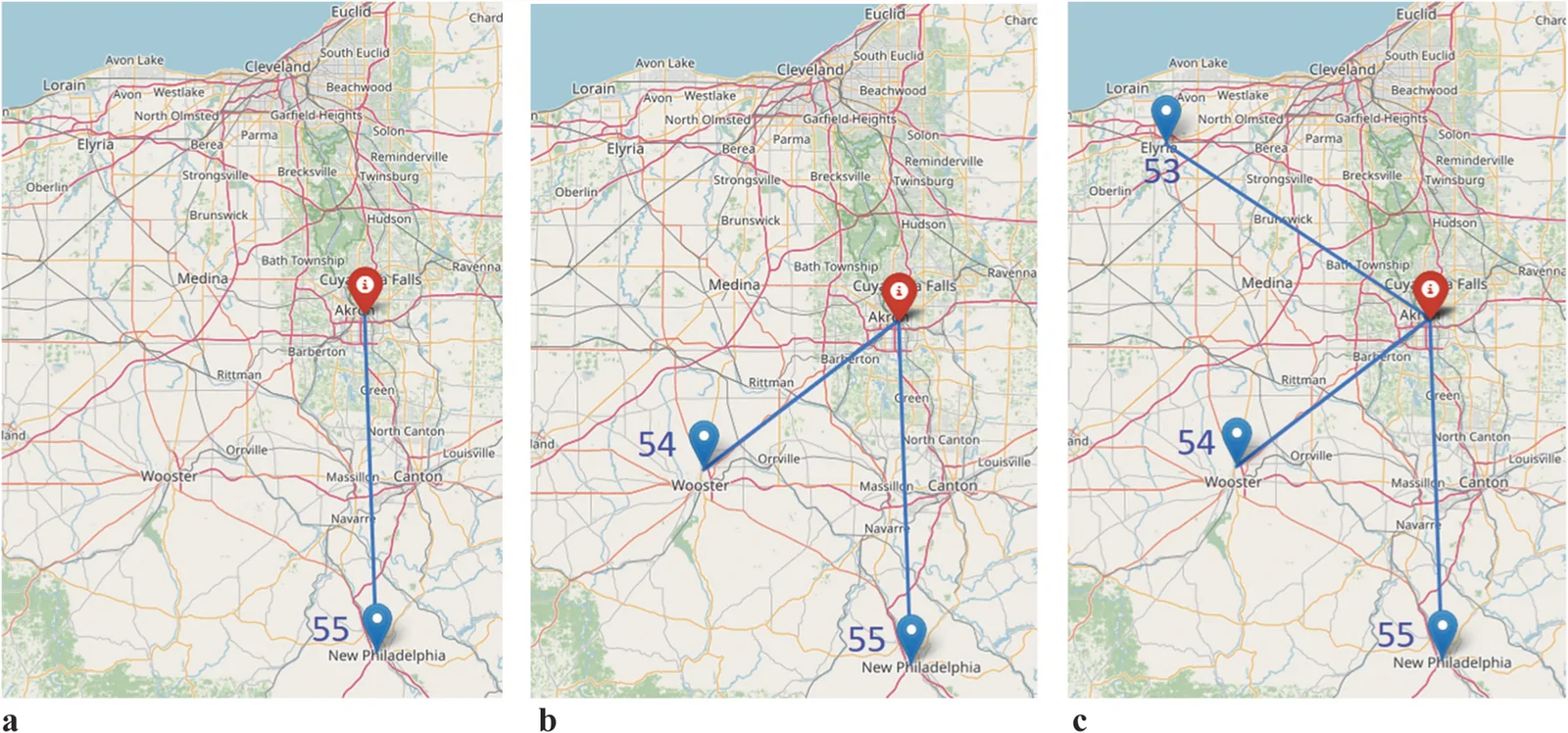
Mobile clinic route based out of Akron Ohio. The red markers indicate the fixed methadone clinic (the starting and stopping location of the mobile route). The blue markers are stops along the route, and the text next to each stop is the number of clients served at that stop. **5a**, **5b**, and **5c **show the one, two, and three vehicle routes respectively

For an understanding of how these routes might look, we consider a clinic operating out of downtown Akron, shown in Fig. 5. The optimal single vehicle route sends a unit 45 min south to New Philadelphia and serves 54.6 demand. When this clinic operates two vehicles, one vehicle operates this same route, while the other travels 45 min southwest to Wooster and serves 54.2 demand from an entirely different population. When operating three vehicles, the optimal solution maintains both of these prior routes and additionally sends a vehicle 49 min northwest to Elyria to serve 52.5 demand. In some instances, there is sufficiently large demand in a small geographic area that multiple routes select stops in areas close together. One such case occurs in Youngstown Ohio, where each route that is added from the same fixed clinic has only one stop. The first route is able to serve 59.1 people at a single location, while for routes two and three the same location is chosen and 54.25 additional clients are served on each of these routes.

## Discussion

The N-SSATS reports that in Ohio, the median methadone clinic reported serving between 201 and 300 outpatient clients per day, with 32% of clinics reporting serving more than 301 clients per day [27]. Owing to the relative scarcity of these existing clinics, there is still high unmet need outside their geographic reach. Our results show the potential of mobile methadone routes in expanding the capacity of existing clinics to reach underserved areas. Our models show an average of 59.7 clients in Ohio can be served with a single mobile methadone clinic route per day under our baseline best estimate of realistic parameter values. These findings are consistent with caseloads by Stewart et al. (2023), who report between 20 and 100 clients at mobile clinics in Philadelphia [38]. Mobile units following optimal routes in Ohio spend on average approximately an hour driving and only stop once, often just to the outskirts of the coverage of their starting clinic. The volume of unmet demand is frequently large enough that a mobile vehicle can stay in one location for its full operating day.

Our sensitivity analysis based on the parameter values demonstrates the importance of estimating operation times, specifically service time, setup time, and how far clients are willing to travel. For small changes, routes tend to serve similar areas of the state, while larger changes may lead to dramatically different optimal routes. Features such as the number of medical professionals able to dispense can affect the service time. For example, if it takes one provider five minutes to serve a client, the average service time with two providers would be two and a half minutes. Other factors affecting service time could include whether counseling or additional services are offered, whether an administrative professional helps check in clients, and the logistics of how methadone is dispensed and clients are served. Service time and setup time may be estimated from data from existing fixed clinic operations and expert opinion, while maximum travel time may be best assessed with surveys of potential clients. After initial operation of a mobile unit, these values can be reassessed and routes adapted. Micro-scale decisions of exactly where within a census tract to add a mobile stop can be influenced by local knowledge, available space, and zoning ordinances. While the stops selected and number of clients served per route change based on the parameter settings, the number of stops made per route often does not.

Nationwide, the demand served is slightly more conservative on average with higher variability than observed in Ohio. This emphasizes the importance of considering each clinic individually. The maximum difference in average clients served per route across metropolitan, micropolitan, small town, and rural based clinics is only 3.5. This shows that it is worthwhile to consider adding mobile routes to clinics regardless of urbanicity. Even though the population density is lower in rural areas, clinic density is also lower, creating large unserved areas. In many of these cases, mobile routes only need to travel to the outskirts of their coverage area to serve a full day’s demand. By comparison, more urban clinics may need to travel farther to escape the coverage of other clinics and reach unserved demand, reducing the total demand that can be served per day. Our search for viable alternative routes shows 42.1% of clinics have no other solutions within 5% of optimality. These findings demonstrate a relatively small amount of flexibility in route planning, further emphasizing the value of this mathematical modeling approach.

The single depot multi-vehicle routing problem can provide service to a higher number of customers spread out over a larger geographic area. In operation, this can either be implemented with multiple vehicles leaving the depot each day or a single vehicle operating on different routes on different days with take-home doses. Providing a single take-home dose for clients provides the same increase in coverage and service as having a second mobile facility without the corresponding cost increase for the physical vehicle and staffing. There is also an improvement in the quality of life for the clients who would then be able to attend the mobile clinic less frequently while still receiving the medication that they need. A common concern in discussion of take-home methadone doses is diversion of the medication [39]. However, records from the organizations that used them during the COVID pandemic indicate that diversion was not a significant problem [40]. The lack of diversion of methadone take-home doses provides a strong justification for the expansion of their use when doing so will improve access to methadone for patients who currently do not have geographic access to this medication.

The high costs of opening and operating a mobile unit implies additional policy changes need to be made to incentivize methadone clinics to meet this demand. Costs incurred under mobile routes include staffing, fuel and fleet maintenance, onboard medication storage and DEA-compliant security systems, communications and EHR technology, and downtime costs while the vehicle is in motion. Revenue is typically generated through Medicaid encounters, take-home dosing reimbursements, and care coordination billing, all of which increasingly allow flexibility for mobile programs. Multi-day take-home dosing reduces per-patient operating costs and spreads fixed costs over a larger patient panel. Mobile units can also reduce cost per patient by operating vehicles with two dosing windows to serve twice as many patients at the same time, as explored in our sensitivity analysis. The drive time to mobile stops and associated setup time would be sufficient to serve on average 34 people per day, which directly translates to a loss of this potential revenue. For mobile clinics to be financially sustainable operations, a change in the funding and reimbursement structures to account for the cost of transit, including the lost revenue from dosing appointments during transit, must be considered.

In addition to refining model parameter values as discussed above, our model is flexible for customizations to address specific goals or concerns. For example, available time windows could be added as constraints to account for known work schedules or commuter patterns. This may be particularly relevant in areas with large commuter populations, which can be identified by the RUCA codes. Introducing weights to the objective function could place greater emphasis on serving demand in rural areas, a priority of some national initiatives [12, 13]. Additional constraints could require mobile units to make a specified number of stops in a day if larger geographic coverage is desired. A modification to the time limitation constraint could account for additional services sometimes provided by mobile clinics, such as counseling, mental health support, and other medical services [13]. Similarly, weights in the objective function could prioritize locations with needs for these other services. Clinics with multiple near-optimal routes as described above can leverage their localized knowledge to choose between them.

This work has explored operational characteristics of mobile methadone maintenance in nonurban areas. We specifically focus on providing daily maintenance treatment to clients. Mobile methadone clinics may serve other purposes, for example client recruitment or educational outreach. Clinics focused on these purposes would face an entirely different set of considerations. Additionally, our purpose is to consider nonurban settings outside of the reach of existing brick and mortar clinics. Mobile clinics may operate in urban environments aiming to reduce travel time of existing clients, future studies may explore operational questions related to these services.

## Limitations and future work

Our work entails a number of limitations. We have made assumptions about reasonable parameter values for operational aspects of mobile clinics because of the newness of the topic and lack of existing scholarly literature. We have assumed a static known demand, whereas realized demand may be variable day-to-day and may shift due to clients starting and stopping methadone treatment. Similarly, service and setup times may vary across patients, days, and geographies. We assume only clients without existing access to methadone will attend the mobile clinic, while in practice some attending a fixed clinic may transfer, potentially reducing the number of new clients served. We have assumed client willingness to attend a mobile clinic is reflected in the maximum travel distance parameter, while other factors such as time of day and availability of transportation may affect it. In our modeling, we assume the fixed clinic under consideration is the only one in the state to run a mobile unit, whereas in practice other clinics may run units which satisfy some demand. Finally, we only consider serving populations with no existing access far from existing clinics, although similar models could be used to plan urban routes for clients with existing access.

In order for clinics to customize this model to establish new mobile routes, they would need to incorporate organization-specific information. Future studies could incorporate clinic-specific data to update the model with localized parameter values. Patient demand and willingness to travel in specific geographic areas could be confirmed or updated by conducting community outreach surveys, key-informant interviews, and environmental scans. Our service time estimates consider only methadone dispensing, whereas clinics may also wish to account for time spent on counseling and other services. The current model estimates travel times through ideal road conditions, while clinics may also include factors such as traffic, inclement weather, construction, or tolls in their drive times. To address traffic delays and congestion, clinics may explore historical traffic patterns, real-time congestion data, appointment schedules, participant density, and insights from previous route performance (e.g. time-to-site and missed appointment rates). Planning buffer times in routes, for example by decreasing the total allowed route length by the magnitude of the expected traffic time, can mitigate unexpected delays and allow for real-time adjustments. Clinics may choose to restrict routes to avoid undesirable features such as having stops on both sides of a major city. These restrictions could be enforced by adding additional constraints to the model. Since the model uses pharmacies as a proxy for mobile stops, exact parking locations would need to be selected. Relevant factors in this selection may include neighborhood acceptance, parking restrictions, and permitting requirements in different jurisdictions. A further economic study could validate the cost efficiency of mobile routes compared to expanding access via other means. Finally, studies could examine the costs and benefits of hybrid solutions that open a new fixed methadone clinic while simultaneously operating a mobile unit out of it.

## Data Availability

All data, code, and supplementary results are available at https://github.com/bonifontea/Mobile-Methadone.
